# Supplementary material for: Genie: the first open-source ISO/IEC encoder for genomic data
Source: Commun Biol. 2024 May 9;7:553. doi: 10.1038/s42003-024-06249-8 (PMC11082222; doi:10.1038/s42003-024-06249-8)
Supplement: Supplementary file 2 — Supplementary Information [file 42003_2024_6249_MOESM2_ESM.pdf]

Supplementary Table 1:
Descriptor Subsequences Semantics

Descriptor	Subsequence	Contents
0	0	Alignment positions, delta encoded
1	0	Flag: Nucleotide sequence is reversed
2	0	Flag: Record is a duplicate
2	1	Flag: Record failed vendor quality check
2	2	Flag: Record contains properly aligned pair
3	0	Flag: Next mismatch belongs to next record
3	1	Mismatch positions in nucleotide sequence, delta encoded
4	0	Mismatch types (substitution, insertion, deletion)
4	1	Substituted nucleotides in mismatches
4	2	Inserted nucleotides in mismatches
5	0	IDs of records with clips
5	1	Type and position in nucleotide sequence of clips
5	2	Softclipped nucleotides with terminators
5	3	Hardclip lengths
6	0	Nucleotide sequences verbatim
7	0	Nucleotide sequence lengths
8	0	Pairing case between nucleotide sequences in a record
8	1	Distance between closely spaced, paired sequences
8	2	Alignment position of nucleotide sequence 1 (same reference, not closely spaced to nucleotide sequence 2)
8	3	Alignment position of nucleotide sequence 2 (same reference, not closely spaced to nucleotide sequence 1)
8	4	Reference sequence of nucleotide sequence 1 (different from nucleotide sequence 2)
8	5	Reference sequence of nucleotide sequence 2 (different from nucleotide sequence 1)
8	6	Alignment position of nucleotide sequence 1 (reference different from nucleotide sequence 2)
8	7	Alignment position of nucleotide sequence 2 (reference different from nucleotide sequence 2)
9	0	Alignment-level quality scores
12	0	Record alignment case (U, P, N, M, I, HM)
13	0	Record group
14	variable	Nucleotide-level quality scores
15	variable	Record identifier tokens

Supplementary Table 1: Descriptor sequences used in Genie as specified in MPEG-G.

Supplementary Table 2: Record Identifier Tokens

Token	Description
DUP	Indicates as first token that string is fully equal to a previous string
DIFF	Indicates as first token that string is delta encoded to a previous string
STRING	Null-terminated string
CHAR	Single ASCII character
DIGITS	32-bit number
DELTA	32-bit number, delta-encoded to other number
MATCH	Indicates token is identical to token in other record at same position
END	Terminator, indicating start of the next record ID

Supplementary Table 2: Tokens used for record ID encoding.

Supplementary Table 3: Genomic Datasets

<table>
<tr><th>ID</th><th>File Name(s)</th><th>Species</th><th>Sequencing Technology</th><th>Coverage</th><th>Origin</th></tr>
<tr><td>01-1..
01-32</td><td>ERR174310_{1|2}.fastq.gz..
ERR174341_{1|2}.fastq.gz</td><td>H. sapiens</td><td>Illumina HiSeq 2000</td><td>n/a</td><td>http://www.ebi.ac.uk/ena/data/view/ERP001775</td></tr>
<tr><td>02-1</td><td>NA12878_S1.bam</td><td>H. sapiens</td><td>Illumina HiSeq 2000</td><td>52.3x</td><td>http://www.ebi.ac.uk/ena/data/view/ERP001960</td></tr>
<tr><td>02-2</td><td>NA12879_S1.bam</td><td>H. sapiens</td><td>Illumina HiSeq 2000</td><td>53.3x</td><td>http://www.ebi.ac.uk/ena/data/view/ERP001960</td></tr>
<tr><td>02-3</td><td>NA12890_S1.bam</td><td>H. sapiens</td><td>Illumina HiSeq 2000</td><td>46.9x</td><td>http://www.ebi.ac.uk/ena/data/view/ERP001960</td></tr>
<tr><td>03</td><td>NA12878.pacbio.
bwa-sw.20140202.bam</td><td>H. sapiens</td><td>PacBio</td><td>8.4x</td><td>ftp://ftp.1000genomes.ebi.ac.uk/vol1/ftp/technical/
Working/20131209_na12878_pacbio/si/NA12878.
Pacbio.bwa-sw.20140202.bam</td></tr>
<tr><td>04-1</td><td>NA12877_S1.bam</td><td>H. sapiens</td><td>Illumina HiSeq 2000</td><td>17.4x</td><td>http://www.ebi.ac.uk/ena/data/view/ERP002490</td></tr>
<tr><td>04-2</td><td>NA12878_S1.bam</td><td>H. sapiens</td><td>Illumina HiSeq 2000</td><td>26.1x</td><td>http://www.ebi.ac.uk/ena/data/view/ERP002490</td></tr>
<tr><td>04-3</td><td>NA12882_S1.bam</td><td>H. sapiens</td><td>Illumina HiSeq 2000</td><td>24.1x</td><td>http://www.ebi.ac.uk/ena/data/view/ERP002490</td></tr>
<tr><td>05</td><td>9827_2#49.bam</td><td>H. sapiens</td><td>Illumina HiSeq 2000</td><td>2.3x</td><td>http://www.ebi.ac.uk/ena/data/view/ERR317482</td></tr>
<tr><td>06</td><td>NA21144.chrom11.
ILLUMINA.bwa.GIH.low
_coverage.20130415.bam</td><td>H. sapiens</td><td>Illumina HiSeq 2000</td><td>7.4x</td><td>ftp://ftp.1000genomes.ebi.ac.uk/vol1/ftp/data/
NA21144/alignment/NA21144.chrom11.ILLUMINA.
Bwa.GIH.low_coverage.20130415.bam</td></tr>
<tr><td>07</td><td>ERR174310_{1|2}.fastq.gz</td><td>H. sapiens</td><td>Illumina HiSeq 2000</td><td>n/a</td><td>http://www.ebi.ac.uk/ena/data/view/ERP001775</td></tr>
<tr><td>08</td><td>*.fastq.gz</td><td>H. sapiens</td><td>PacBio</td><td>n/a</td><td>http://datasets.pacb.com/2014/Human54x/fast.html</td></tr>
<tr><td>09-1</td><td>sample-2-10_sorted.bam</td><td>H. sapiens</td><td>Ion Torrent PGM</td><td>274.0x</td><td>http://www.ebi.ac.uk/ena/data/view/ERX276880</td></tr>
<tr><td>09-2</td><td>sample-2-11_sorted.bam</td><td>H. sapiens</td><td>Ion Torrent PGM</td><td>238.9x</td><td>http://www.ebi.ac.uk/ena/data/view/ERX276881</td></tr>
<tr><td>09-3</td><td>sample-2-12_sorted.bam</td><td>H. sapiens</td><td>Ion Torrent PGM</td><td>225.4x</td><td>http://www.ebi.ac.uk/ena/data/view/ERX276882</td></tr>
<tr><td>11</td><td>SRR1238539.fastq.gz</td><td>H. sapiens</td><td>Ion Torrent PGM</td><td>n/a</td><td>ftp://ftp.ddbj.nig.ac.jp/ddbj_database/dra/fastq/
SRA096/SRA096885/
SRX517292/SRR1238539.fastq.bz2</td></tr>
<tr><td>12</td><td>NA12878_V2.5_Robot
_2_R{1|2}.fastq.gz</td><td>H. sapiens</td><td>Illumina HiSeq X Ten</td><td>n/a</td><td>https://s3-ap-southeast-2.amazonaws.com/kccg-x10-
Truseq-nano-v2.5-na12878/NA12878_V2.5_
Robot_2_R{1|2}.fastq.gz</td></tr>
<tr><td>13</td><td>Ecoli_R7_NONI.fastq.gz</td><td>E. coli</td><td>ONT MinION</td><td>n/a</td><td>http://www.ebi.ac.uk/ena/data/view/ERX593919</td></tr>
<tr><td>14</td><td>Ecoli_R73.fastq.gz</td><td>E. coli</td><td>ONT MinION</td><td>n/a</td><td>http://www.ebi.ac.uk/ena/data/view/ERX593921</td></tr>
<tr><td>15</td><td>SRR1284073.fastq.gz</td><td>E. coli</td><td>PacBio</td><td>n/a</td><td>ftp://ftp.ddbj.nig.ac.jp/ddbj_database/dra/sralite/
ByExp/litesra/SRX/SRX533/SRX533603</td></tr>
<tr><td>16</td><td>MiSeq_Ecoli_DH10B
_110721_PF.bam</td><td>E. coli</td><td>Illumina MiSeq</td><td>447.8x</td><td>ftp://webdata:webdata@ussd-ftp.illumina.com/
Data/SequencingRuns/DH10B/
MiSeq_Ecoli_DH10B_110721_PF.bam</td></tr>
<tr><td>18</td><td>SRR327342.bam</td><td>S. cerevisiae</td><td>Illumina/Solexa
Genome Analyzer</td><td>n/a</td><td>ftp://ftp.ddbj.nig.ac.jp/ddbj_database/dra/fastq/
SRA043/SRA043851/SRX089128</td></tr>
<tr><td>19</td><td>dm3PacBio.bam</td><td>D.
melanogaster</td><td>PacBio</td><td>81.2x</td><td>http://bergmanlab.ls.manchester.ac.uk/data/
Tracks/dm3/dm3PacBio.bam</td></tr>
<tr><td>24</td><td>SRR870667.fastq.gz</td><td>T. cacao</td><td>Illumina Genome
Analyzer IIx</td><td>n/a</td><td>http://www.ncbi.nlm.nih.gov/sra/SRX288435</td></tr>
<tr><td>25</td><td>SRR554369_{1|2}.fastq.gz</td><td>P. aeruginosa</td><td>Illumina Genome
Analyzer IIx</td><td>n/a</td><td>ftp://ftp.ddbj.nig.ac.jp/ddbj_database/dra/fastq/
SRA058/SRA058002/SRX181937</td></tr>
<tr><td>26</td><td>simulation.1.homoINDELs.
HomoCEUsnps.reads2.fq
.sam.samelength.bam</td><td>H. sapiens</td><td>ART
(mimicking Illumina)</td><td>n/a</td><td>n/a</td></tr>
<tr><td>31</td><td>ERR174324.aln_bowtie2.sorted.
Dupmark.rg.realn.recal.bam</td><td>H. sapiens</td><td>Illumina HiSeq 2000</td><td>15.2x</td><td>n/a</td></tr>
</table>

Supplementary Table 3: Properties and origins of datasets.

ID	File Name(s)	Species	Sequencing Technology	Coverage	Origin
32	NA12878-Rep-1_S1_L001_2_R{1|2}_001.fastq.trimmed.gz	H. sapiens	Illumina NovaSeq 6000	n/a	n/a
33	Phix1_NoIndex_L001_R{1|2}_001.fastq.gz	Phi X 174	Illumina NovaSeq 6000	n/a	n/a
20-1	MH0001_081026_clean.{1|2}.fq.gz	n/a	Illumina Genome Analyzer IIx	n/a	ftp://ftp.sra.ebi.ac.uk/vol1/ERA000/ERA000116/Fastq/MH0001_081026_clean.{1|2}.fq.gz
20-2	MH0002_081203_clear.{1|2}.fq.gz	n/a	Illumina Genome Analyzer IIx	n/a	ftp://ftp.sra.ebi.ac.uk/vol1/ERA000/ERA000116/Fastq/MH0002_081203_clear.{1|2}.fq.gz
20-3	MH0003_081203.clean.{1|2}.fq.gz	n/a	Illumina Genome Analyzer IIx	n/a	ftp://ftp.sra.ebi.ac.uk/vol1/ERA000/ERA000116/Fastq/MH0003_081203.clean.{1|2}.fq.gz
21	G15511.HCC1143_BL.1.bam	H. sapiens	Illumina HiSeq 2000	56.0x	https://cghub.ucsc.edu/datasets/benchmark_download.html
22	G15511.HCC1143.1.bam	H. sapiens	Illumina HiSeq 2000	46.5x	https://cghub.ucsc.edu/datasets/benchmark_download.html
23	HCC1954.mix1.n80t20.bam	H. sapiens	Illumina HiSeq 2000	25.9x	https://cghub.ucsc.edu/datasets/benchmark_download.html
10	K562_cytosol_LID8465_TopHat_v2.bam	H. sapiens	Illumina HiSeq 2000	n/a	http://www.ebi.ac.uk/arrayexpress/files/E-MTAB-1728/K562_ cytosol_LID8465_TopHat_v2.bam
30	K562_cytosol_LID8465_GEM_v3.bam	H. sapiens	Illumina HiSeq 2000	n/a	n/a
34	ERR174310.aln.sort.dupmark.rg.recal.bam	H. sapiens	Illumina HiSeq 2000	n/a	n/a
35	ERR174310.mult.sorted.bam	H. sapiens	Illumina HiSeq 2000	n/a	n/a
36-1	ERR1638068_{1|2}.fastq.gz	H. sapiens	BGISEQ-500	42x	https://www.ebi.ac.uk/ena/data/view/ERR1638068
36-2	ERR1638069_{1|2}.fastq.gz	H. sapiens	BGISEQ-500	42x	https://www.ebi.ac.uk/ena/data/view/ERR1638069
37	NA12878-Rep-1_S1_L001_2.Aln.sort.dupmark.rg.bam	H. sapiens	Illumina NovaSeq 6000	n/a	n/a
38	ERR174310.chr9.rn.bam	H. sapiens	Illumina HiSeq 2000	13,6	n/a
39	ERR174310.chr9_1.fq.gz	H. sapiens	Illumina HiSeq 2000	n/a	n/a
40	ERR174310.chr9_2.fq.gz	H. sapiens	Illumina HiSeq 2000	n/a	n/a
41	ERR174310.unmapped_1.fq.gz	H. sapiens	Illumina HiSeq 2000	n/a	n/a
42	ERR174310.unmapped_2.fq.gz	H. sapiens	Illumina HiSeq 2000	n/a	n/a
44	ERR174310.chr9_1.sub_2m.fq.gz	H. sapiens	Illumina HiSeq 2000	n/a	n/a
45	ERR174310.chr9_1.sub_20k.fq.gz	H. sapiens	Illumina HiSeq 2000	n/a	n/a
46	ERR174310.chr9_1.sub_200k.fq.gz	H. sapiens	Illumina HiSeq 2000	n/a	n/a
48	G15511.HCC1143_BL.1.chr9.rn.bam	H. sapiens	Illumina HiSeq 2000	62,42	n/a
49	G15511.HCC1143_BL.1.chr9_1.fq.gz	H. sapiens	Illumina HiSeq 2000	n/a	n/a
50	G15511.HCC1143_BL.1.chr9_2.fq.gz	H. sapiens	Illumina HiSeq 2000	n/a	n/a
53	G15511.HCC1143_BL.1.chr9_1.sub_2m.fq.gz	H. sapiens	Illumina HiSeq 2000	n/a	n/a
54	G15511.HCC1143_BL.1.chr9_1.sub_20k.fq.gz	H. sapiens	Illumina HiSeq 2000	n/a	n/a
55	G15511.HCC1143_BL.1.chr9_1.sub_200k.fq.gz	H. sapiens	Illumina HiSeq 2000	n/a	n/a
ONT-1	HG002.fastq.gz	H. sapiens	ONT Promethion	n/a	https://labs.epi2me.io/askenazi-kit14-2022-12/
ONT-2	HG002.bam	H. sapiens	ONT Promethion	n/a	https://labs.epi2me.io/askenazi-kit14-2022-12/

Supplementary Table 3: Properties and origins of datasets.

ID	Study Accession	Sample Accession	Experiment Accession	Run Accession
01	PRJEB3246	SAMEA1531956	ERX1504 [56-87]	ERR1743[10-41]
02	PRJEB3381	SAMEA157361[7-8]	ERX1688[36;48]	ERR194147
05	PRJEB1682	SAMEA2056087	ERX290630	ERR317482
07	PRJEB3246	SAMEA1531956	ERX150456	ERR174310
09	PRJEB4193	SAMEA21449[23;38;43]	ERX27688[0-2]	ERR3035[39-41]
13	PRJEB7385	SAMEA2785537	ERX593919	ERR637420
14	PRJEB7385	SAMEA2785537	ERX593921	ERR637419
24	SRP004925	SAMN01881290	SRX288435	SRR870667
36	PRJEB15427	SAMEA4443138	ERX170840[3-4]	ERR163806[8-9]

Available accession codes.

Supplementary Table 4: Optimized Set of Parameters

Descriptor	Subsequence	Transform	Transform Parameter	Coding Order	Symbol Size	Subsymbol Size	Subsymbol Transform	Binarization	Binarization Parameter
0	0	1	0	2 0 -	1 32 -	1 32 -	0 0 -	0 2 -	- - -
1	0	1	0	2 1 -	1 1 -	1 1 -	0 1 -	0 1 -	- 1 -
2	0	0	0	0 - -	1 - -	1 - -	0 - -	0 - -	- - -
2	1	0	0	1 - -	1 - -	1 - -	1 - -	1 - -	1 - -
2	2	0	0	0 - -	1 - -	1 - -	0 - -	0 - -	- - -
3	0	0	0	2 - -	1 - -	1 - -	0 - -	0 - -	- - -
3	1	2	255	0 1 0	8 8 32	8 8 32	0 1 0	2 1 2	- 255 -
4	0	0	0	1 - -	2 - -	2 - -	1 - -	1 - -	2 - -
4	1	0	0	1 - -	3 - -	3 - -	1 - -	1 - -	4 - -
4	2	0	0	2 - -	3 - -	3 - -	0 - -	1 - -	4 - -
5	0	0	0	0 - -	32 - -	32 - -	0 - -	0 - -	- - -
5	1	0	0	2 - -	4 - -	4 - -	1 - -	1 - -	8 - -
5	2	0	0	2 - -	3 - -	3 - -	1 - -	1 - -	5 - -
6	0	0	0	2 - -	3 - -	3 - -	1 - -	1 - -	4 - -
7	0	1	0	1 0 -	1 32 -	1 32 -	1 0 -	1 2 -	1 - -
8	0	1	0	2 2 -	1 3 -	1 3 -	0 1 -	0 1 -	- 6 -
8	1	3	255	0 0 -	8 16 -	8 16 -	0 0 -	1 2 -	255 - -
8	2	3	255	1 0 -	8 32 -	2 32 -	1 0 -	1 0 -	255 - -
8	3	1	0	1 0 -	1 32 -	1 32 -	1 0 -	1 0 -	1 - -
8	4	3	255	0 0 -	8 16 -	8 16 -	0 0 -	2 2 -	- - -
8	5	3	255	0 0 -	8 16 -	8 16 -	0 0 -	2 2 -	- - -
8	6	1	0	0 0 -	1 32 -	1 32 -	0 0 -	0 0 -	- - -
8	7	3	255	0 0 -	8 32 -	8 32 -	0 0 -	1 0 -	255 - -
9	0	3	255	1 1 -	8 8 -	8 8 -	1 1 -	2 1 -	- 255 -
12	0	0	0	1 - -	3 - -	3 - -	1 - -	1 - -	6 - -
14	2	0	0	2 - -	8 - -	8 - -	1 - -	1 - -	255 - -
14	3	0	0	1 - -	8 - -	8 - -	1 - -	1 - -	255 - -

Supplementary Table 4: Parameters found during optimization. Multiple values in the same column correspond to the transformed descriptor subsequences generated during the sequence transformation.

Supplementary Table 5: Compression Loss Through Static Sequence Transformation

		Compressed Size (Bytes)		
**Encoding**	**Item**	**Dynamic Conf.**	**Static Conf.**	**Size Ratio**
Low Latency	01-1	70092398	70160007	1,001
Global Assembly	01-1	157331725	157552232	1,001
Low Latency	32	55909170	55927424	1,000
Global Assembly	32	50476217	50595832	1,002
Local Assembly	02-1	109425305	109557150	1,001
Reference-Based	02-1	101174649	101275082	1,001
Local Assembly	37	44190804	44338928	1,003
Reference-Based	37	37329463	37440621	1,003

Supplementary Table 5: The compressed size of the first ten access units of selected items. Compared are the sizes for a globally optimized, constant parameter set versus dynamic parameter set that is optimized individually for each access unit. The last column is the ratio between both sizes.

Supplementary Note 1:
Commands Used during Simulations

Genie (for unaligned data), version 9bdd63 (transcoding not included in timings):

```
# transcode FASTQ to MPEG-G records
Genie transcode-FASTQ | \
    --input-file input_1.FASTQ \
    --input-suppl-file input_2.FASTQ \
    --output-file transcoded.mgrec \
    --threads ${num_threads}
```

```
# global assembly encode
Genie run \
    --threads ${num_threads} \
    --input-file transcoded.mgrec \
    --output-file encoded.mgb
```

```
# low latency encode
Genie run \
    --low-latency \
    --threads ${num_threads} \
    --input-file transcoded.mgrec \
    --output-file encoded.mgb
```

```
# Decode
Genie run \
    --threads ${num_threads} \
    --input-file encoded.mgb \
    --output-file decoded.mgrec
```

DSRC 2.0:

```
# Encode
dsrc c \
    -t ${num_threads} \
    input_1.FASTQ encoded_1.dsrc
```

```
# Decode
dsrc d \
    -t ${num_threads} \
    encoded_1.dsrc decoded_1.FASTQ
```

pigz (gzip) 2.7:

```
# Encode
pigz \
    --stdout input_1.FASTQ \
    --processes ${num_threads} \
    > encoded_1.gzip
```

```
# Decode
pigz \
    --decompress \
    --processes ${num_threads} \
    --stdout encoded_1.gzip \
    > decoded_1.FASTQ
```

Mstcom 8eb5ab2:
Note: this tool does not support the compression of record identifiers. We added the size of the compressed identifiers obtained in the Genie (global assembly) experiments to the results of these tools to enable a fair comparison with the other tools.

```
# Encode
Mstcom e \
    -t ${num_threads} \
    -i input_1.FASTQ \
    -f input_2.FASTQ \
    -o input_1.Mstcom
```

```
# Decode
Mstcom d \
    -i input_1.Mstcom \
    -o decoded_1.FASTQ
```

PgRC 1.2:

Note: this tool does not support the compression of record identifiers. We added the size of the compressed identifiers obtained in the Genie (global assembly) experiments to the results of these tools to enable a fair comparison with the other tools.

```
# Encode
PgRC \
    -i input_1.FASTQ input_2.FASTQ \
    -t ${num_threads} \
    input_1.pgrc
```

```
# Decode
PgRC \
    -t ${num_threads} \
    -d input_1.pgrc
```

Genozip 15.0.4:

```
# Encode
genozip \
    -@ ${num_threads} \
    --no-test input_1.FASTQ \
    -o input_1.genozip
```

```
# Decode
genozip \
    -@ ${num_threads} \
    -d input_1.genozip \
    -o input_1.FASTQ
```

Cleaning input BAM files from unsupported alignments and optional tags:

```
cat input.bam | \
samtools sort \
    -n \
    -@ 6 \
    -O SAM | \
Genie transcode-sam\
    -i "-.sam" \
    -o "-.mgrec" \
    -r reference.fa \
    -c \
    -f \
    -w ./tmp_directory \
    -t ${num_threads} | \
Genie transcode-sam \
    -i "-.mgrec" \
    -o "-.sam" \
    -f \
    -r reference.fa | \
samtools sort \
    -@ 6 \
    -O BAM \
> cleaned.bam
```

Converting BAM to uncompressed SAM before benchmarks:

```
samtools view \
    -@ ${num_threads} \
    -h \
    ${bam_file} \
    -o ${sam_file}
```

BAM (samtools 1.18):

```
# Encode
samtools view \
    -@ ${num_threads} \
    -b \
    -h \
    input.sam\
    -o encoded.bam
```

```
# Decode
samtools view \
    -@ ${num_threads} \
    -h \
    encoded.bam \
    -o decoded.sam
```

Cram 3.1 (samtools 1.18):

```
# Encode
samtools view \
    -@ ${num_threads} \
    -O CRAM \
    -h \
    -T reference.FASTA
    input.sam \
    --output-fmt-option version=3.1 \
    -o encoded.cram
```

```
# Decode
samtools view \
    -@ ${num_threads} \
    -h \
    encoded.cram \
    -o decoded.sam
```

Genozip 15.0.4:

```
# Encode
genozip \
    -@ ${num_threads} \
    input.sam \
    --no-test \
    --reference reference.FASTA \
    -o encoded.genozip -f
```

```
# Decode
genozip \
    -@ ${num_threads} \
    -d encoded.genozip \
    --reference reference.FASTA \
    -o decoded.sam
```

DeeZ 1.9:

```
# Encode
deez \
    --threads ${num_threads} \
    --reference reference.FASTA \
    input.sam\
    --output encoded.deez
```

```
# Decode
deez \
    --threads ${num_threads} \
    --reference reference.FASTA \
    encoded.deez \
    --output decoded.sam\
    --header
```

Genie (for aligned data):

```
# Transcoding SAM to mgrec:
Genie transcode-sam \
    --threads ${num_threads} \
    --input-file input.sam\
    --output-file transcoded.mgrec \
    -r reference.FASTA \
    -w ./tmp_dir \
    -c
```

```
# Reference based encoding
Genie run \
    --threads ${num_threads} \
    --input-file transcoded.mgrec \
    --output-file encoded.mgb \
    --input-ref-file reference.FASTA
```

```
# Local assembly encoding
Genie run \
    --threads ${num_threads} \
    --input-file transcoded.mgrec \
    --output-file encoded.mgb
```

```
# Decoding
Genie run \
    --threads ${num_threads} \
    --input-file encoded.mgb \
    --output-file transcoded.mgrec
```

Measurement of execution times with gnu/time 1.9:

```
time --verbose --output time.txt ${command}
```

Measurement of memory usage with memory-profiler 0.57.0:

```
mprof run -o memory.log ${command}
```
