# Supplementary material for: Genie: the first open-source ISO/IEC encoder for genomic data
Source: Commun Biol. 2024 May 9;7:553. doi: 10.1038/s42003-024-06249-8 (PMC11082222; doi:10.1038/s42003-024-06249-8)
Supplement: Supplementary file 3 — Description of Additional Supplementary Files [file 42003_2024_6249_MOESM3_ESM.pdf]

Description of Additional Supplementary Files

File name: Supplementary Data 1

Description: The results from the experiments and source data behind the graphs in the paper.
