# Supplementary material for: Genie: the first open-source ISO/IEC encoder for genomic data
Source: Commun Biol. 2024 May 9;7:553. doi: 10.1038/s42003-024-06249-8 (PMC11082222; doi:10.1038/s42003-024-06249-8)
Supplement: Supplementary file 5 — Reporting Summary [file 42003_2024_6249_MOESM5_ESM.pdf]

nature portfolio

Corresponding author(s): Fabian Müntefering
Mikel Hernaez
Jan Voges

Last updated by author(s): Apr 14, 2024

Reporting Summary

Nature Portfolio wishes to improve the reproducibility of the work that we publish. This form provides structure for consistency and transparency in reporting. For further information on Nature Portfolio policies, see our Editorial Policies and the Editorial Policy Checklist.

Statistics

For all statistical analyses, confirm that the following items are present in the figure legend, table legend, main text, or Methods section.

n/a	Confirmed	
☐	☒	The exact sample size (n) for each experimental group/condition, given as a discrete number and unit of measurement
☐	☒	A statement on whether measurements were taken from distinct samples or whether the same sample was measured repeatedly
☒	☐	The statistical test(s) used AND whether they are one- or two-sided<br>*Only common tests should be described solely by name; describe more complex techniques in the Methods section.*
☒	☐	A description of all covariates tested
☒	☐	A description of any assumptions or corrections, such as tests of normality and adjustment for multiple comparisons
☒	☐	A full description of the statistical parameters including central tendency (e.g. means) or other basic estimates (e.g. regression coefficient) AND variation (e.g. standard deviation) or associated estimates of uncertainty (e.g. confidence intervals)
☒	☐	For null hypothesis testing, the test statistic (e.g. F, t, r) with confidence intervals, effect sizes, degrees of freedom and P value noted<br>*Give P values as exact values whenever suitable.*
☒	☐	For Bayesian analysis, information on the choice of priors and Markov chain Monte Carlo settings
☒	☐	For hierarchical and complex designs, identification of the appropriate level for tests and full reporting of outcomes
☒	☐	Estimates of effect sizes (e.g. Cohen's d, Pearson's r), indicating how they were calculated

Our web collection on statistics for biologists contains articles on many of the points above.

Software and code

Policy information about availability of computer code

Data collection	No genomic data was collected, software was developed to process already existing data.
Data analysis	Our code is available at https://github.com/MueFab/genie. It is also made available at https://doi.org/10.5281/zenodo.10967397<br>Other software used and their version is described in supplementary material F.

For manuscripts utilizing custom algorithms or software that are central to the research but not yet described in published literature, software must be made available to editors and reviewers. We strongly encourage code deposition in a community repository (e.g. GitHub). See the Nature Portfolio guidelines for submitting code & software for further information.

Data

Policy information about availability of data

All manuscripts must include a data availability statement. This statement should provide the following information, where applicable:

- Accession codes, unique identifiers, or web links for publicly available datasets
- A description of any restrictions on data availability
- For clinical datasets or third party data, please ensure that the statement adheres to our policy

All sequencing data used for our benchmarks is available in the MPEG-G Genomic Information Database (https://mpeg.chiariglione.org/standards/MPEG-G/genomic-information-representation/MPEG-G-genomic-information-database-4) on request. A subset of the data has also been made available for direct download

from various online repositories by their respective original authors. The table in supplementary material C provides more details on the online availability of each dataset. The numeric experimental results used to generate the figures are included in supplementary material E.

Research involving human participants, their data, or biological material

Policy information about studies with human participants or human data. See also policy information about sex, gender (identity/presentation), and sexual orientation and race, ethnicity and racism.

Reporting on sex and gender	No human participants.
Reporting on race, ethnicity, or other socially relevant groupings	No human participants.
Population characteristics	No human participants.
Recruitment	No human participants.
Ethics oversight	No human participants, no ethically critical research.

Note that full information on the approval of the study protocol must also be provided in the manuscript.

Field-specific reporting

Please select the one below that is the best fit for your research. If you are not sure, read the appropriate sections before making your selection.

☒ Life sciences ☐ Behavioural & social sciences ☐ Ecological, evolutionary & environmental sciences

For a reference copy of the document with all sections, see nature.com/documents/nr-reporting-summary-flat.pdf

Life sciences study design

All studies must disclose on these points even when the disclosure is negative.

Sample size	The sample size was chosen to include multiple data sources, species and sequencing technologies. No further statistical claims are made that require a specific sample size.
Data exclusions	Aligned genomic records with features unsupported in the current version of our software (e.g. half-aligned read pairs) were transformed or removed removed before the analysis with any software (our own and third party), as described in the manuscript.
Replication	We did not discover any inconsistencies in the results during subsequent runs on parts of the datasets (as part of the continuing development and improvement of the software).
Randomization	No randomization is required for evaluation of software.
Blinding	No blinding is required for the evaluation of software.

Reporting for specific materials, systems and methods

We require information from authors about some types of materials, experimental systems and methods used in many studies. Here, indicate whether each material, system or method listed is relevant to your study. If you are not sure if a list item applies to your research, read the appropriate section before selecting a response.

Materials & experimental systems

n/a	Involved in the study	
☒	☐	Antibodies
☒	☐	Eukaryotic cell lines
☒	☐	Palaeontology and archaeology
☒	☐	Animals and other organisms
☒	☐	Clinical data
☒	☐	Dual use research of concern
☒	☐	Plants

Methods

n/a	Involved in the study	
☒	☐	ChIP-seq
☒	☐	Flow cytometry
☒	☐	MRI-based neuroimaging

Plants

Seed stocks	No plants used
Novel plant genotypes	No plants used
Authentication	No plants used
